# Efficient Reverse Transcription Using Locked Nucleic Acid Nucleotides towards the Evolution of Nuclease Resistant RNA Aptamers

**DOI:** 10.1371/journal.pone.0035990

**Published:** 2012-04-25

**Authors:** Lucile Crouzier, Camille Dubois, Stacey L. Edwards, Lasse H. Lauridsen, Jesper Wengel, Rakesh N. Veedu

**Affiliations:** 1 School of Chemistry and Molecular Biosciences, The University of Queensland, St Lucia, Brisbane, Queensland, Australia; 2 Institute Polytechnique LaSalle Beauvais, Beauvais, France; 3 The Novo Nordisk Foundation Center for Biosustainability, Scion DTU, Hørsholm, Denmark; 4 Nucleic Acid Center, Department of Physics and Chemistry, University of Southern Denmark, Odense, Denmark; University of Helsinki, Finland

## Abstract

**Background:**

Modified nucleotides are increasingly being utilized in the *de novo* selection of aptamers for enhancing their drug-like character and abolishing the need for time consuming trial-and-error based post-selection modifications. Locked nucleic acid (LNA) is one of the most prominent and successful nucleic acid analogues because of its remarkable properties, and widely explored as building blocks in therapeutic oligonucleotides. Evolution of LNA-modified RNA aptamers requires an efficient reverse transcription method for PCR enrichment of the selected RNA aptamer candidates. Establishing this key step is a pre-requisite for performing LNA-modified RNA aptamer selection.

**Methodology:**

In this study three different reverse transcriptases were investigated towards the enzymatic recognition of LNA nucleotides. Both incorporation as well as reading capabilities of the LNA nucleotides was investigated to fully understand the limitations of the enzymatic recognition.

**Conclusions:**

We found that SuperScript® III Reverse Transcriptase is an efficient enzyme for the recognition of LNA nucleotides, making it a prime candidate to be used in *de novo* selection of LNA containing RNA aptamers

## Introduction

Nucleic acid-based therapeutic approaches have gained significant interests in recent years towards the development of drugs against many diseases. Nucleic acid aptamers [1]–[5] are an emerging class of therapeutic molecules, and a chemically-modified RNA aptamer Macugen® (Pegaptanib sodium), an inhibitor of vascular endothelial growth factor (VEGF) has been approved for the treatment of age related macular degeneration (AMD) [6], [7]. To improve the pharmacokinetic properties of aptamers e.g., nuclease resistance, chemically-modified nucleotides have to be used. Aptamers are normally generated by an *in vitro* evolution process referred to as SELEX (Systematic Evolution of Ligands by EXponential enrichment) [8]–[10]. Application of modified nucleotides in SELEX processes is rather limited because of their poor substrate properties to various polymerases.

**Figure 1:**
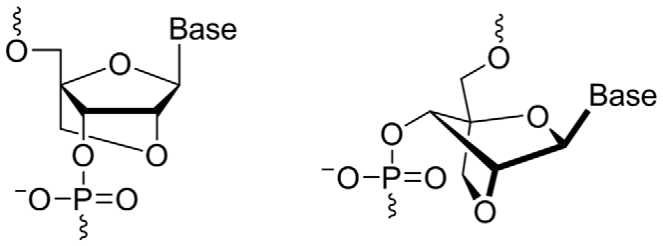
Structural representations of LNA monomers.

Locked Nucleic Acid (LNA) is one of the most prominent and successful nucleic acid analogues because of its remarkable properties, and it is widely explored as building blocks in therapeutic oligonucleotides [11]–[14]. LNA nucleotides are generally considered to be RNA mimicking molecules in which the ribose sugar moiety is locked by an oxymethylene linkage connecting the C2′ and C4′ carbon atoms, imposing conformational restriction to adopt C3′-endo/N -type furanose conformation (Figure 1) [15]–[17]. We have previously reported the enzymatic recognition capabilities of LNA nucleotides by DNA polymerases towards their applicability in the evolution of DNA aptamers [18]–[22]. Evolution of LNA-modified RNA aptamers requires an efficient reverse transcription method for PCR enrichment of the selected RNA aptamer candidates. Establishing this key step is a pre-requisite for performing LNA-modified RNA aptamer selection. Herein, for the first time, we report an efficient reverse transcription using LNA nucleotides.

## Results and Discussion

**Figure 2:**
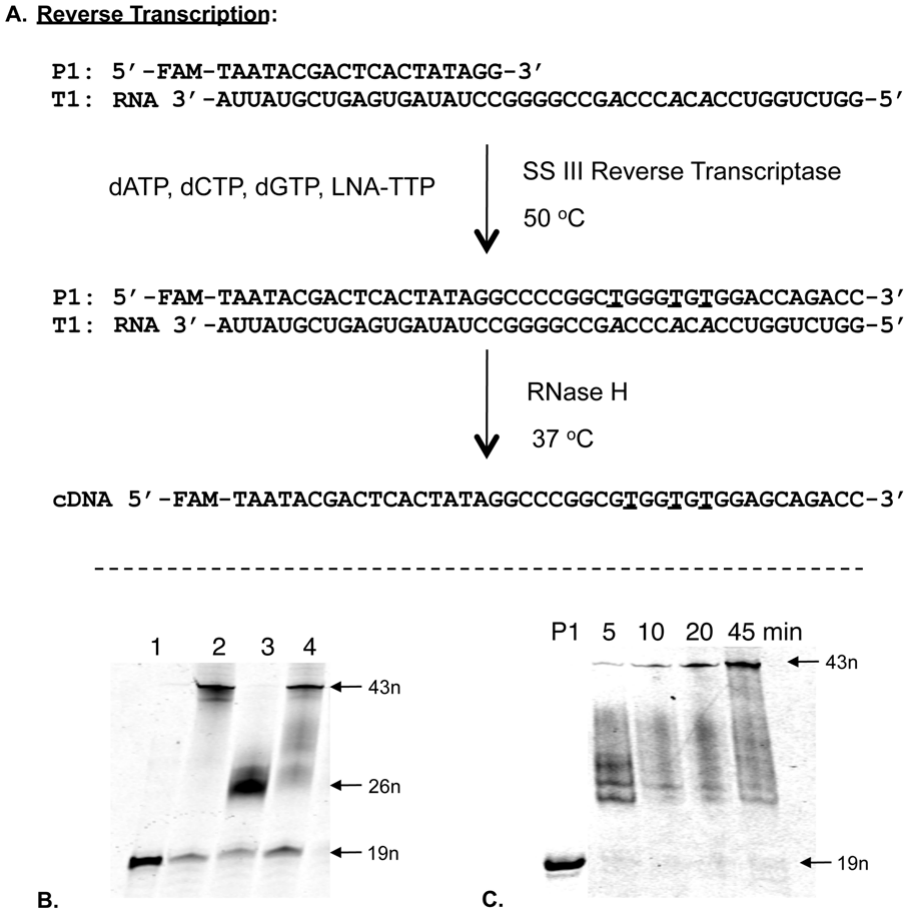
Enzymatic incorporation of LNA-T nucleotides by SuperScript® III Reverse Transcriptase. (A) Primer and template sequences. (B) Gel electrophoresis of incorporation LNA-T, lane 1: Primer, 19 nt; lane 2: Positive control using all four natural dNTPs; lane 3: Negative control reaction with only three natural nucleotides (dCTP, dATP, dGTP); lane 4: Incorporation of LNA-T (dCTP, dATP, dGTP, LNA-TTP in the mixture). (C) Gel electrophoresis of incorporation LNA-T at 5–45 minute incubation time points, lane P1: Primer. LNA nucleotides are underlined and the incorporation sites in the template RNA are italicized.

First, we investigated LNA-TTP (LNA-T-5′-triphosphate) as a substrate of three different reverse transcriptases; SuperScript® III, M-MuLV (Moloney Murine Leukemia Virus) and AMV (Avian Myo-blastosis Virus). The designed RNA template T1 (43 nt, Figure 2A) has three sites of incorporations for LNA-T nucleotides. In all experiments, we included positive and negative control reactions, in parallel to the one involving LNA-TTP in place of dTTP. The positive control reaction contained all four natural dNTPs, resulting in full-length cDNA products. The negative control reaction mixture lacked the nucleoside triphosphate similar to the LNA nucleotide to be tested for incorporation, and thus the extension was expected to stop at the nucleotide prior to the first site of LNA incorporation. The resulting cDNA products were resolved by denaturing polyacrylamide gel electrophoresis for which the primer DNA P1 (19 nt, Figure 2A) was 5′-FAM-labelled.

The experiments demonstrated that SuperScript® III Reverse Transcriptase efficiently incorporated LNA-T nucleotides, encoded by RNA nucleotides of the template strand, and are capable of producing full-length cDNA products in good yield (Figure 2B, lane 4 & C). To check the fidelity and accuracy of the reaction products, we performed polymerase chain reaction (PCR) using the cDNA product as the template followed by cloning and sequencing. We have previously reported the PCR involving LNA-nucleotides [20]. The sequencing chromatogram clearly matched with the expected PCR product sequences confirming the accuracy of the reverse transcription by SuperScript® III (Figure S1, supporting information). The yield of full-length cDNA containing LNA nucleotides was greater at 45 minutes of incubation (Figure 2C). M-MuLV Reverse Transcriptase was also able to produce the full-length cDNA, however, it was not as efficient as SuperScript® III Reverse Transcriptase (Figure S2B lane 4, supporting information). AMV Reverse Transcriptase afforded the full-length cDNA product in only very low yield (Figure S2C lane 4, supporting information).

**Figure 3:**
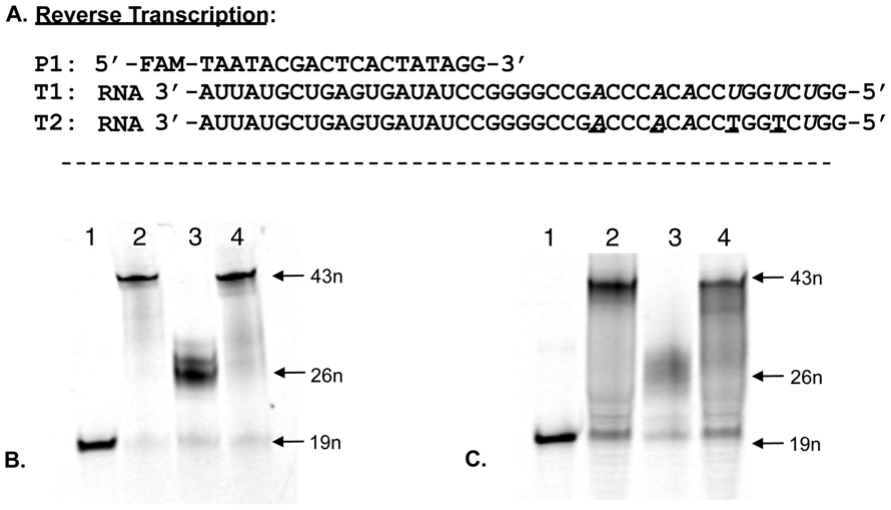
Enzymatic incorporation of LNA-T, LNA-A, dT/LNA-T and dA nucleotides using RNA and LNA/RNA templates. (A) Primer and template sequences. (B) Gel electrophoresis of LNA-T and LNA-A incorporation on a natural RNA template T1, lane 1: Primer, 19 nt; lane 2: Positive control using all four natural dNTPs; lane 3: Negative control reaction with only two natural nucleotides (dCTP, dGTP); lane 4: Incorporation of LNA-T and LNA-A (dCTP, dGTP, LNA-ATP, LNA-TTP in the mixture). (C) Gel electrophoresis of dT/LNA-T and dA nucleotide incorporation using a LNA-A and LNA-T-modified RNA template T2, lane 1: Primer, 19 nt; lane 2: Positive control using all four natural dNTPs; lane 3: Negative control reaction with only three natural nucleotides (dCTP, dATP, dGTP); lane 4: Incorporation of LNA-T (dCTP, dATP, dGTP, LNA-TTP in the mixture). LNA nucleotides are underlined and the incorporation sites in the template RNA are italicized.

Next, we performed an experiment using both LNA-TTP and LNA-ATP in one reaction by replacing dTTP and dATP. The designed RNA template sequence T1 directs incorporation of first three LNA-T nucleotides followed by three LNA-A nucleotides in the resulting cDNA product. The results show that SuperScript® III Reverse Transcriptase efficiently incorporated both LNA-T and LNA-A nucleotides and furnished the full-length cDNA product in excellent yield (Figure 3B, lane 4). In this experiment, both AMV and M-MuLV Reverse Transcriptases failed to afford the expected full-length cDNA product.

**Figure 4:**
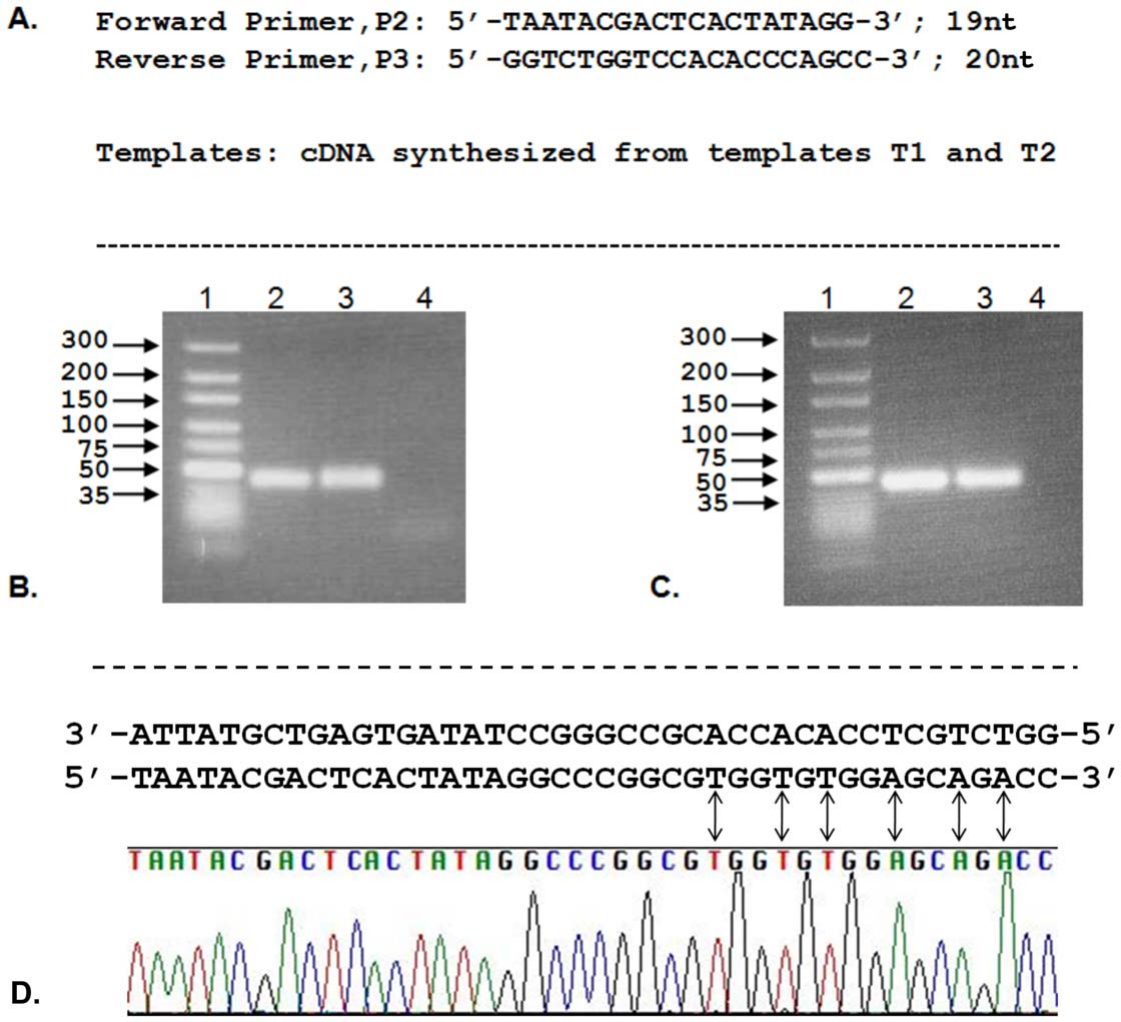
PCR amplification using the cDNA template generated by reverse transcription by SuperScript® III Reverse Transcriptase. (A) Primer and template sequences; (B) Agarose gel electrophoresis image of PCR using the cDNA template containing LNA-T generated by SuperScript® III Reverse Transcriptase, lane 1: DNA size markers in base-pairs, lane 2: PCR using the cDNA with natural DNA nucleotides, lane 3: PCR using cDNA template with LNA-T (43 nt), lane 4: PCR without using a template (negative control); (C) Agarose gel electrophoresis image of PCR using the cDNA template with natural DNA nucleotides and LNA-T nucleotides generated from an LNA-modified RNA template T2 by SuperScript® III Reverse Transcriptase, lane 1: DNA size markers in base-pairs, lane 2: PCR using the cDNA with natural DNA nucleotides, lane 3: PCR using cDNA template with LNA-T, lane 4: PCR without using a template (negative control); (D) PCR product analysis by cloning and sequencing, PCR product obtained from Figure 4C (lane 2) aligned with the sequencing chromatogram. PCR product containing DNA-T and DNA-A at the expected positions of the LNA nucleotides of the initial RNA template is matched with a double headed arrow as identified in the sequencing chromatogram.

The first enzymatic step involved in RNA aptamer selection by SELEX is to convert the selected RNA aptamer sequences to a complementary DNA for PCR amplification. Encouraged by the initial results, we then performed reverse transcription experiments using an LNA-modified RNA template T2. The designed RNA template has four LNA nucleotides with two LNA-A nucleotides first followed by two LNA-T nucleotides opposite to which the desired DNA nucleotides have to be incorporated. We have also conducted one reaction to check the incorporation of LNA-T nucleotides opposite to LNA-A nucleotides of the template sequence by replacing dTTP with LNA-TTP in the dNTP mixture. These experiments revealed that SuperScript® III Reverse Transcriptase efficiently transcribed the LNA nucleotides of the template RNA strand by incorporating DNA nucleotides and afforded the cDNA product in very good yield (Figure 3C, lane 2). PCR amplification followed by cloning and subsequent sequencing analysis was performed to verify the cDNA product. The resulting sequence chromatogram clearly matched with the expected PCR product sequences confirming the accuracy of the reverse transcription using LNA-modified RNA template (Figure 4C, lane 2 & D). Furthermore, SuperScript® III Reverse Transcriptase was able to incorporate LNA-T nucleotides opposite to LNA-A nucleotides of the template strand and furnished an LNA-containing full-length cDNA product in reasonably good yield (Figure 3C, lane 4). AMV and M-MuLV Reverse Transcriptases were less efficient to afford the full-length cDNA products compared to the Super Script III Reverse Transcriptase.

In summary, we have demonstrated that SuperScript® III Reverse Transcriptase is an efficient enzyme to recognize LNA nucleotides and to synthesize a complementary DNA copy from an LNA-modified RNA template. These findings are vital and pave the way towards applying LNA nucleotides in RNA aptamer selection by *in vitro* evolution processes.

## Materials and Methods

### Materials

DNA sequences were purchased from Integrated DNA Technologies (Coralville, Iowa). SuperScript® III Reverse Transcriptase, RNase OUT™ (Ribonuclease inhibitor) and RNase H were purchased from Invitrogen Australia (Mulgrave, Victoria). M-MuLV and AMV Reverse Transcriptases were purchased from New England biolabs (supplied by Genesearch Australia). Phusion DNA polymerase was purchased from Finnzymes (supplied by Genesearch Australia).

### Reverse transcription

The 5′-end labelled primer DNA was annealed with the templates by mixing primer and template in a molar ratio of 1∶2 and heating to 80°C for 3 min followed by slow cooling to 37°C.

### For SuperScript® III Reverse Transcriptase

The reaction mixtures were prepared in a total volume of 20 µL by adding 4 µL of 5× First Strand buffer, 4 µL of MgCl_2_ (25 mM), 1.5 µL of dithiothreitol (DTT, 100 mM), 1.5 µL of dNTPs/LNA-NTPs (2.5 mM of dNTP and 5 mM for LNA-NTP) mixture, 1.5 µL of a solution containing 5′-FAM-labelled primer-template complex (5∶10 pmol ratio), 5 µL of two times distilled water, 1 µL RNase OUT™ (40 U/µL) and 1 µL of SuperScript® III Reverse Transcriptase (250 U/µL). The reaction mixture was gently vortexed and incubated for 45 min at 50°C. Positive and negative control reactions were stopped after 3 minutes of incubation at 50°C as prolonged incubation resulted in product degradation. Then, the reaction mixtures were heated at 80°C for 5 minutes to inactivate the enzyme. To the resulting mixture, 1 µL of RANase H (5 U/µL) was added and incubated for 1 h at 37°C. The reactions were then quenched by the addition of half volume of loading buffer. Analysis of the products was by performing 13% 7 M urea denaturing polyacrylamide gel electrophoresis in the presence of TBE buffer (100 mM Tris, 90 mM boric acid, 1 mM EDTA).

### For M-MulV and AMV Reverse Transcriptase

In this case, the reaction mixtures were prepared in a total volume of 20 µL by adding 2 µL of 10× RT buffer, 4 µL of dNTPs/LNA-NTPs mixture (prepared the same way as mentioned above), 2 µL of a solution containing 5′-FAM-labelled primer-template complex (5∶10 pmol ratio), 10 µL of two times distilled water, 1 µL RNase OUT™ (40 U/µL) and 1 µL of M-MuLV (200 U/µL) or AMV (10 U/µL) Reverse Transcriptase. The reaction mixtures were gently vortexed and incubated for 1 h at 42°C. Positive and negative control reaction was stopped after 3 minutes of incubation at 42°C. Then the reaction mixtures were heated at 90°C for 10 min to inactivate the enzyme. To the resulting mixture, 1 µL of RNase H (5 U/µL) was added and incubated for 1 h at 37°C. The reactions were then quenched by the addition of half volume of loading buffer and analysed as discussed above.

### General procedure for polymerase chain reactions

The PCR reaction mixture was prepared in a total volume of 50 µL by adding 10 µL 5× Phusion HF buffer (included in the Phusion DNA polymerase kit), 4 µL of dNTPs (400 µM), 28 µL of two times distilled water, 1.5 µL of forward primer (50 µM), 1.5 µL of reverse primer (50 µM), 5 µL of template (0,7 µM) stemming from the reverse transcription and 0,5 µL of Phusion DNA polymerase (250 U/µL). The reaction mixtures were gently vortexed and then amplified using a thermal cycler (S1000™ Thermal cycler, Bio-Rad). A 25-cycle PCR consisted of denaturation at 98°C for 10 second, annealing at 55°C for 15 seconds and extension at 72°C for 25 seconds. After the polymerase reactions, gel-loading buffer (included in Ultra Low DNA size marker kit from Fermentas, supplied by VWR Australia) was added (1.5 µL) and the products were analysed by 4% agarose gel electrophoresis followed by UV-photography.

### Cloning and Sequencing

Purified PCR products (50 ng) were cloned into pCR-Blunt (Invitrogen) according to the manufacturer's instructions. Plasmid DNA was extracted using QIAprep (Qiagen) and sequenced by the Australian Genome Research Facility (AGRF, Brisbane, Australia) to validate sequence changes.

## Supporting Information

Figure S1 Sequence analysis of PCR product obtained from a cDNA template containing LNA-T by cloning and sequencing. Position of LNA-T in the expected PCR product is matched with a double headed arrow as identified in the sequencing chromatogram.
(TIF)

Figure S2 Enzymatic incorporation of LNA-T nucleotides. A. Primer and template sequences. B. Gel electrophoresis of incorporation LNA-T by M-MuLV Reverse Transcriptase, lane 1: Primer, 19n; lane 2: Positive control using all four natural dNTPs; lane 3: Negative control reaction with only three natural nucleotides (dCTP, dATP, dGTP); lane 4: Incorporation of LNA-T (dCTP, dATP, dGTP, LNA-TTP in the mixture). B. Gel electrophoresis of incorporation LNA-T by AMV Reverse Transcriptase, lane 1: Primer, 19 nt; lane 2: Positive control using all four natural dNTPs; lane 3: Negative control reaction with only three natural nucleotides (dCTP, dATP, dGTP); lane 4: Incorporation of LNA-T (dCTP, dATP, dGTP, LNA-TTP in the mixture).
(TIF)
