# Supplementary figures and images for: Efficient Reverse Transcription Using Locked Nucleic Acid Nucleotides towards the Evolution of Nuclease Resistant RNA Aptamers
Source: PLoS One. 2012 Apr 25;7(4):e35990. doi: 10.1371/journal.pone.0035990 (PMC3338489; doi:10.1371/journal.pone.0035990)

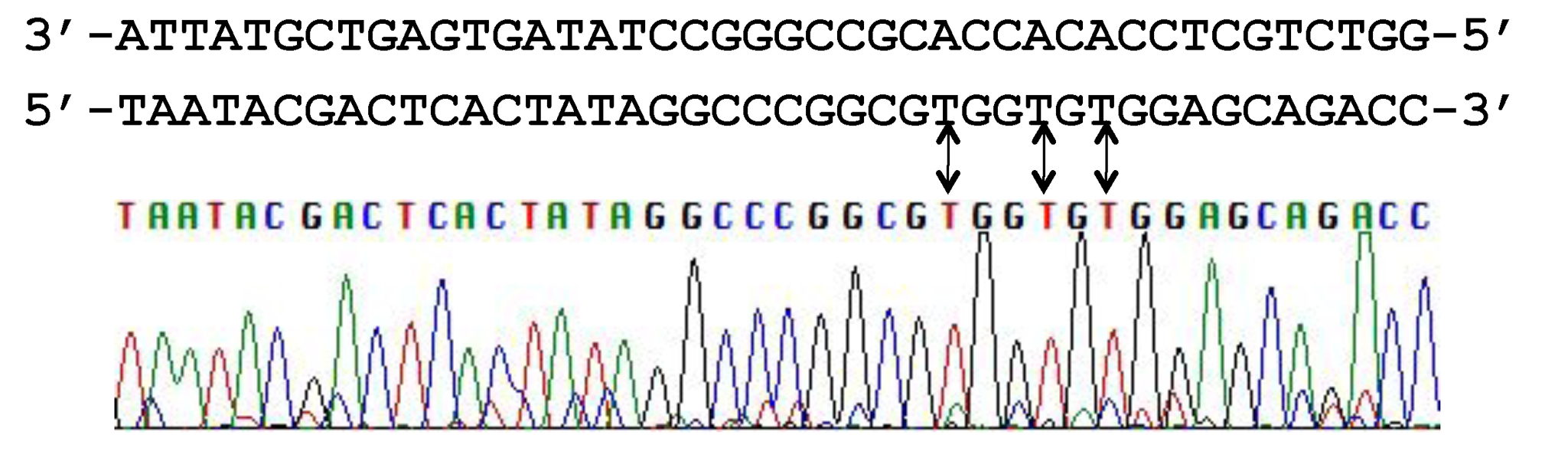

Supplement: Figure S1 — Sequence analysis of PCR product obtained from a cDNA template containing LNA-T by cloning and sequencing. Position of LNA-T in the expected PCR product is matched with a double headed arrow as identified in the sequencing chromatogram. (TIF) [file pone.0035990.s001.tif]

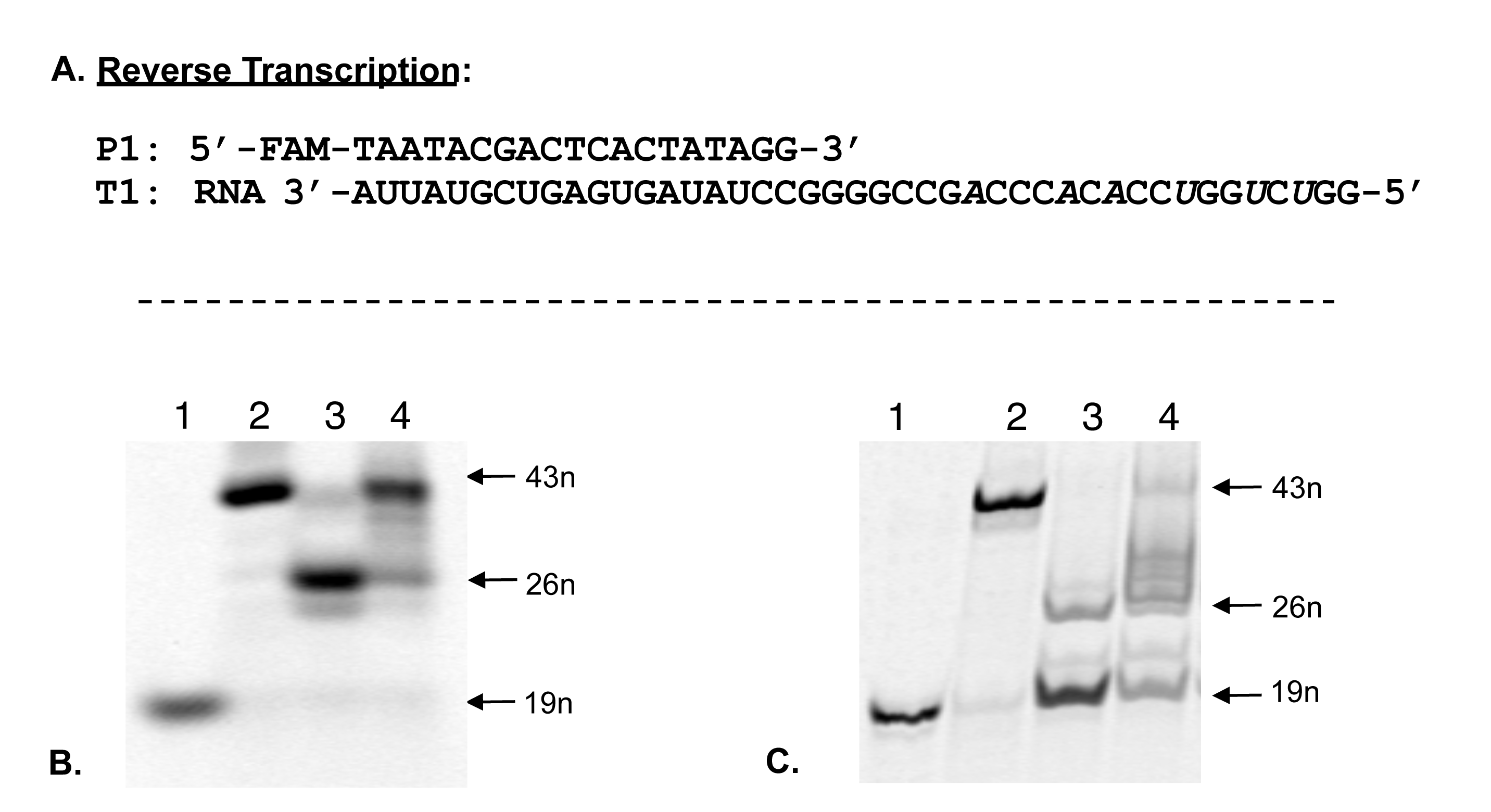

Supplement: Figure S2 — Enzymatic incorporation of LNA-T nucleotides. A. Primer and template sequences. B. Gel electrophoresis of incorporation LNA-T by M-MuLV Reverse Transcriptase, lane 1: Primer, 19n; lane 2: Positive control using all four natural dNTPs; lane 3: Negative control reaction with only three natural nucleotides (dCTP, dATP, dGTP); lane 4: Incorporation of LNA-T (dCTP, dATP, dGTP, LNA-TTP in the mixture). B. Gel electrophoresis of incorporation LNA-T by AMV Reverse Transcriptase, lane 1: Primer, 19 nt; lane 2: Positive control using all four natural dNTPs; lane 3: Negative control reaction with only three natural nucleotides (dCTP, dATP, dGTP); lane 4: Incorporation of LNA-T (dCTP, dATP, dGTP, LNA-TTP in the mixture). (TIF) [file pone.0035990.s002.tif]
